# Development of a Sinitic Clubroot Differential Set for the Pathotype Classification of *Plasmodiophora brassicae*

**DOI:** 10.3389/fpls.2020.568771

**Published:** 2020-08-31

**Authors:** Wenxing Pang, Yue Liang, Zongxiang Zhan, Xiaonan Li, Zhongyun Piao

**Affiliations:** ^1^College of Horticulture, Shenyang Agricultural University, Shenyang, China; ^2^College of Plant Protection, Shenyang Agricultural University, Shenyang, China

**Keywords:** *Plasmodiophora brassicae*, clubroot, pathotype, Sinitic clubroot differential set, resistance

## Abstract

*Plasmodiophora brassicae*, which is known for its broad genetic diversity for virulence, is the causal agent of clubroot disease of *Brassica* crops worldwide. Studies on pathotype characterization with four differential hosts according to Williams’ classification system showed the predominance of pathotype 4 in China. However, the genetic variability within pathotype 4 complicates the breeding of durable clubroot-resistant (CR) cultivars. Herein, a Sinitic clubroot differential (SCD) set was developed using a set of eight differential inbred lines of Chinese cabbage with known or novel *CR* genes. The presence of immense diversity within pathotype 4 of Williams’ system was verified, and 11 pathotypes were characterized using the developed SCD system. The scalability and practicability of the system was further confirmed with a subset of 95 field isolates from different *Brassica* crops and different regions of China and Korea. Sixteen pathotypes were detected from 132 field isolates, named Pb1 to Pb16, respectively. Among them, Pb1 and Pb4 were prevalent in diverse *Brassica* crops in the southern and northern regions of China. Pb12, Pb13, Pb14, and Pb16 showed area-specific distribution. The SCD set developed herein will provide important genetic resources for pathogenicity studies of *P. brassicae* and for CR breeding in Chinese cabbage and other *Brassica* crops.

## Introduction

The emergence and rapid spread of clubroot disease, caused by the soil-borne obligate plant pathogen *Plasmodiophora brassicae* Woronin, has become one of the most serious diseases of cruciferous crops worldwide (Dixon, 2009). *Brassica* crops, including Chinese cabbage (*B. rapa*), cabbage (*B. oleracea*), oilseed rape (*B. napus*), radish (*R. sativus*), and tuber mustard (*B. juncea*) are infected by clubroot disease in most cultivation areas (Chai et al., 2014), and 3.2–4.0 million ha of *Brassica* crops are affected annually in China (Wang et al., 2011). Breeding of clubroot-resistant (CR) cultivars is believed to be the most effective and environment-friendly strategy for clubroot management. Many CR cultivars of Chinese cabbage and canola have been released in Japan, China, Korea, and Canada. However, pathotype- or race-specific CR genes and pathotype variation causes CR cultivars to lose their resistance within 3 to 4 years (Kuginuki et al., 1999; Strelkov et al., 2016). Therefore, the accurate estimation of the pathotypes of field isolates by differential hosts for *P. brassicae* could provide critical information for the selection and cultivation of possible CR cultivars with specific *CR* genes in different areas. Furthermore, differential hosts will provide important materials for clubroot resistance breeding to improve clubroot resistance (Zhang et al., 2017). The pathotypes can be determined by assaying their infection spectra on a set of differential host plants carrying different resistance genes (Inukai et al., 1994). Specifically, the mining of CR germplasm and identification of *CR* genes in *B. rapa* are not only important for the breeding of CR cultivars but also provide possibilities to develop sets of differential hosts for *P. brassicae*.

To study the genetic variation of *P. brassicae*, several pathotype classification systems have been developed in the past decades based on serial host sets of *Brassica* species showing resistance or partial resistance. Williams’ classification system, one of the commonly used systems, was proposed based on two hosts of *B. oleracea* (“Badger shipper” and “Jersey Queen”) and two hosts of *B. napobrassica* (“Laurentian” and “Wilhelmsburger”) by Williams (1966). Theoretically, this system could differentiate 16 pathotypes or races. Among them, pathotype 4 was widely distributed in China, Japan and Korea (Kuginuki et al., 1999; Shen et al., 2009; Ji et al., 2013; Kim et al., 2016). However, some *P. brassicae* isolates which were identified as the same pathotype by Williams’ system, caused different disease severities in Chinese cabbage (Kim et al., 2016), suggesting that this system may not be suitable for pathotype classification and clubroot resistance breeding. Subsequently, a European clubroot differential (ECD) set, including five hosts each from *B. rapa* (ssp. *rapifera* line aaBBCC, ssp. *rapifera* line AAbbCC, ssp. *rapifera* line AABBcc, ssp. *rapifera* line AABBCC and ssp. *pekinensis* cv. “Granaat”), *B. napus* (“Nevin,” “Dc119,” “Giant Rape” selection, New Zealand resistant rape and var. napobrassica “Wilhemsburger”), and *B. oleracea* (var. *capitata* cv. “Badger Shipper,” var. *capitata* cv. “Bindsachsener,” var. *capitata* cv. “Jersey Queen,” var. *capitata* cv. “Septa” and var. *acephala* subvar. *laciniata* cv. “Verheul”), was developed (Buczacki et al., 1975). Some et al. (1996) developed another differential set with three lines of *B. napus* (“Nevin,” “Wilhelmsburger,” and “Brutor”), which classified 20 field collections of *P. brassicae* into five groups. Recently, the Canadian clubroot differential (CCD) set, containing 13 differential hosts (*B. rapa* ssp. *rapifera* line AAbbCC, *B. rapa* var. *pekinensis* “Granaat,” Brassica napus “Nevin,” *B. napus* “Giant Rape” selection, B. napus New Zealand resistant rape, B. napus *var*. napobrassica “Wilhemsburger,” Brassica oleracea *var*. capitata “Badger Shipper,” Brassica oleracea var. *capitata* cv. Jersey Queen, B. napus “Brutor,” B. napus *var*. napobrassica “Laurentian,” B. napus “Westar,” B. napus “Mendel” and B. napus “45H29”), was introduced, and 106 field isolates were classified into 17 unique pathotypes in Canada (Strelkov et al., 2018). However, a couple of limitations regarding the host sets used in these classification systems should be highlighted. For example, 3 common hosts of Williams’ system included in the ECD and CCD sets did not show uniform resistance or susceptibility to some isolates of *P. brassicae* (Kuginuki et al., 1999). Moreover, the ECD system has a complicated system of nomenclature, resulting in inconvenient understanding or wide acceptance (Strelkov and Hwang, 2014; Strelkov et al., 2018). In addition, none of the earlier *P. brassicae* classification systems of Williams (1966), ECD (Buczacki et al., 1975), Some et al. (1996) and CCD (Strelkov et al., 2018) identified the putative clubroot resistance genes in their differential hosts.

As more CR genes are being identified in *B. rapa*, several commercial CR hybrids of Chinese cabbage have been used for local pathogen classification, such as “Noranggimjang,” “CR-Cheongrok,” “Degao CR1016,” and “Akimeki” in Korea (Kim et al., 2016), and “CR Kanko,” “CR Kukai 65,” “CR Ryutoku,” “CR Utage 70,” and “CR W-1116” in Japan (Kuginuki et al., 1999). However, commercial F_1_ hybrids of Chinese cabbage used for clubroot differentiation are not always available, leading to the limitation of their utility in the future. Furthermore, some *CR* genes are inherited as complete dominant (Hatakeyama et al., 2013; Pang et al., 2018), while some of them are quantitative manner (Chen et al., 2013; Pang et al., 2014). In such cases, F_1_ hybrids are not recommended to be used as hosts to differentiate pathotypes of *P. brassicae*. In addition, the genetic base and *CR* genes are not clear in most of the hosts used in these clubroot differentiation sets, except for the hosts ECD01 to ECD04 (Hirani et al., 2018).

Currently, clubroot caused by *P. brassicae* is predominantly prevalent in China (Chai et al., 2014). Cruciferous crops, including vegetable and oilseed crops, are widely cultivated with different cultivation modes in diverse climatic conditions in China. For example, a rice-rape rotation system is widely adopted. Cultivation modes and the diverse climatic circumstances may provide a favorable environment for the prevalence of clubroot disease and pathogenic variation of *P. brassicae*. Therefore, a suitable clubroot differential system is required for pathological studies and for the improvement of clubroot resistance.

In this study, we aimed to: (i) develop a set of differential hosts for the stable characterization of *P. brassicae* pathotypes using inbred lines of Chinese cabbage, (ii) classify the pathotypes of *P. brassicae* in China using the developed SCD, and (iii) analyze the distribution characteristics of *P. brassicae* in China. In addition, we discuss the utilization of these differential hosts.

## Materials and Methods

### Plant Materials

Four hosts from the Williams’ classification system, including “Badger Shipper,” “Jersey Queen,” “Laurentian,” and “Wilhelmsburger” were used in this study. The above four differential hosts and eleven CR inbred lines of Chinese cabbage were infected with 37 isolates for the selection of possible hosts to develop a new clubroot differential system, named the Sinitic clubroot differential (SCD) set (**Table 1**). An inbred line of Chinese cabbage, “BJN3-1,” was used as a clubroot-susceptible control. Isolate Collection and Plant Inoculation

**Table 1:** Clubroot resistance genes detection of Chinese cabbage inbred lines used in this study.

Accession number	Known CR gene/genes detection	Detail information	Source
CR-26	*Crr1*	Landraces	SYAU^a^
222	*CRa*	Chen et al., 2013	SYAU^a^
CR-20	*Crr1, Crr4*	Landraces	SYAU^a^
CR-77	*CRa, Crr1*	Pang et al., 2018	SYAU^a^
CR-75	*CRa, Crr2*	Pang et al., 2018	SYAU^a^
85-74	*CRd*	Pang et al., 2018	SYAU^a^
CR-73	*Crr3, Crr4*	Pang et al., 2018	SYAU^a^
CR-096	Novel CR gene/genes	Landraces	SYAU^a^
CR-179	*CRa*	Landraces	SYAU^a^
CR-189	*CRa*	Landraces	SYAU^a^
CR-190	*CRa*	Landraces	SYAU^a^
BJN3-1	*None*	Pang et al., 2018	SYAU^a^

^a^Shenyang Agricultural University.

In total, 132 field isolates collected from the infected roots of Chinese cabbage, canola, broccoli, or wild mustard from China and Korea were used in this study (**Table S1**). Of these, 37 (numbers 1–37) were used to inoculate to 4 hosts from Williams’ set (1966) and 12 Chinese cabbage inbred lines (**Table 1**), for the development of the clubroot differential set. The remaining 95 isolates were used to inoculate eight selected CR hosts (**Table 2**, H01–H08) to validate and evaluate the practicability, stability and extensibility of the developed SCD system. To accurately evaluate the pathotypes of *P. brassicae*, the resistance tests lasted from 2015 to 2019. All these materials were planted in 72-well multi-pots with 3 replications and maintained in a greenhouse at 20°C to 25°C under a 16-h photoperiod until inoculation with *P. brassicae*. Each replication contained 24 plants.

**Table 2:** The hosts and virulence pattern of Sinitic clubroot differential (SCD) set for *Plasmodiophora brassicae*.

Accession number	Host Code	Pb1	Pb2	Pb3	Pb4	Pb5	Pb6	Pb7	Pb8	Pb9	Pb10	Pb11	Pb12	Pb13	Pb14	Pb15	Pb16
CR-096	H01	−	−	−	−	−	−	−	−	−	+	−	−	−	−	−	−
CR-20	H02	−	−	−	−	−	−	−	−	+	+	+	−	−	+	+	+
CR-26	H03	−	−	−	−	−	−	+	+	+	+	+	−	−	−	+	−
CR-77	H04	−	−	−	−	−	+	−	+	+	+	+	−	+	−	+	+
222	H05	−	−	−	+	+	+	−	+	−	−	+	−	−	+	+	+
CR-75	H06	−	−	−	+	−	−	−	−	−	−	+	+	−	+	−	+
85-74	H07	−	−	+	−	−	−	−	−	−	−	−	−	+	−	−	−
CR-73	H08	−	+	−	−	−	−	−	−	−	−	−	−	−	−	−	−
BJN3-1	H09	+	+	+	+	+	+	+	+	+	+	+	+	+	+	+	+

All field isolates were propagated with the “BJN3-1” line under controlled environments, and fresh galls were stored at −20°C for further use. Preparation of the resting spores of *P. brassicae* was according to Williams (1966) with minor modifications. Briefly, after the galls were ground in sterile distilled water with a homogenizer, the mixtures were filtered through 8 layers of cheesecloth. The resting spores were collected by centrifugation at 2,500*g* and quantified with a hemocytometer (Neubauer improved, Marienfeld, Germany). The concentration of resting spores was adjusted to 1 × 10^7^/ml, and 1 ml of the suspension was inoculated to the 5-day-old seedlings of each host. The potting mixture (Fanyu, Shenyang, China) was kept moist until 6 weeks after *P. brassicae* inoculation.

### Disease Index Evaluation

Disease symptoms were evaluated at 6 weeks after *P. brassicae* inoculation. After the roots were thoroughly washed, disease symptoms were scored as follows: 0, no symptoms; 1, a few small clubs on the lateral roots; 2, larger clubs on the lateral roots or small clubs on the main roots; and 3, large galls both on the lateral and main roots. Disease index (DI) was further calculated according to the formula DI = Σ[nw] × 100/3T, where n is the number of plants in each score class, w is the disease score (0 to 3), and T is the total number of plants tested (Chaube and Singh, 1990). If the disease incidence and DI of “BJN3-1” was lower than 90% and 80, respectively, or the disease incidence of CR hosts was intermediate, the resistance tests were repeated until the susceptible controls were fully diseased. The resistance was determined if the mean DI was lower than 25 and its associated 95% confidence interval did not overlap 50% (Some et al., 1996; Strelkov et al., 2016); otherwise, susceptibility was determined.

### Genotyping CR Inbred Lines at CR Loci

To understand the genetic background of CR hosts, the published CR locus/gene including *CRa*, *CRc*, *CRd*, *Crr1*, *Crr2*, *Crr3*, and *Crr4* were genotyped using CR locus-/gene-linked markers and newly designed markers. *CRa* gene full-length amplification primers were designed using Primer3 (v. 0.4.0) according to the sequence of *CRa* (Koressaar and Remm, 2007; Ueno et al., 2012; Untergasser et al., 2012). *CRc* was detected using forward primer B50-C9-FM and reverse B50-RV (Matsumoto et al., 2012); *CRd* was detected using marker yau376 and yau389 (Pang et al., 2018); *Crr1*, *Crr2*, *Crr3*, and *Crr4* were detected using marker BSA7, BRMS-096, OPC11-2S, and BRMS-125, respectively (Suwabe et al., 2003; Saito et al., 2006; Suwabe et al., 2006; Hatakeyama et al., 2013). All detected CR locus/gene and their linked marker sequences were given in **Table S2**.

### Selection and Verification of the SCD Set

The similarity in the resistance response of all CR lines to 37 isolates of *P. brassicae* classified by Williams’ set was analyzed using MEGA version 5 (Tamura et al., 2011). The virulence pattern of resistance was used for the UPGMA phylogeny tree construction (Lee and Palci, 2015). The phylogeny tree was constructed using MEGA based on the maximum composite-likelihood model (Tamura et al., 2011). The bootstrap method was used for testing the phylogeny with 1,000 bootstrap replications. Redundant hosts showing highly similar or identical disease response patterns to other hosts were removed. On combining these data with the known CR gene detection data, the representative hosts were selected and used to develop the SCD system (**Table 2**). To verify the developed SCD set, a subset of 95 field isolates collected from China and Korea were tested (38–132, **Table S1**). Subsequently, the phylogenetic tree was constructed using 132 isolates of *P. brassicae* in this study.

## Results

### Pathotype Differentiation Based on Williams’ Classification System

The pathotypes of 37 field isolates collected from different *Brassica* crops in China were classified using Williams’ classification system. However, all the 4 hosts showed unstable response to some isolates even though susceptible controls were completely infected. To accurately classify the pathotypes of these isolates, the infection tests were repeated several times through the years of 2015 to 2017 until at least 1 host was completely infected and stable data sets for each host were obtained. Finally, the pathotype for each field isolate was determined by assigning a threshold value, as described in the methods. Accordingly, 37 field isolates were classified into 4 pathotypes, including pathotype 2 with 6 isolates, pathotype 4 with 28 isolates, pathotype 7 with 2 isolates, and pathotype 11 with only 1 isolate (**Supplementary Table S3**).

### Reaction Patterns of CR Inbred Lines to 4 Different Pathotypes of *P. brassicae*

**Figure 1:**
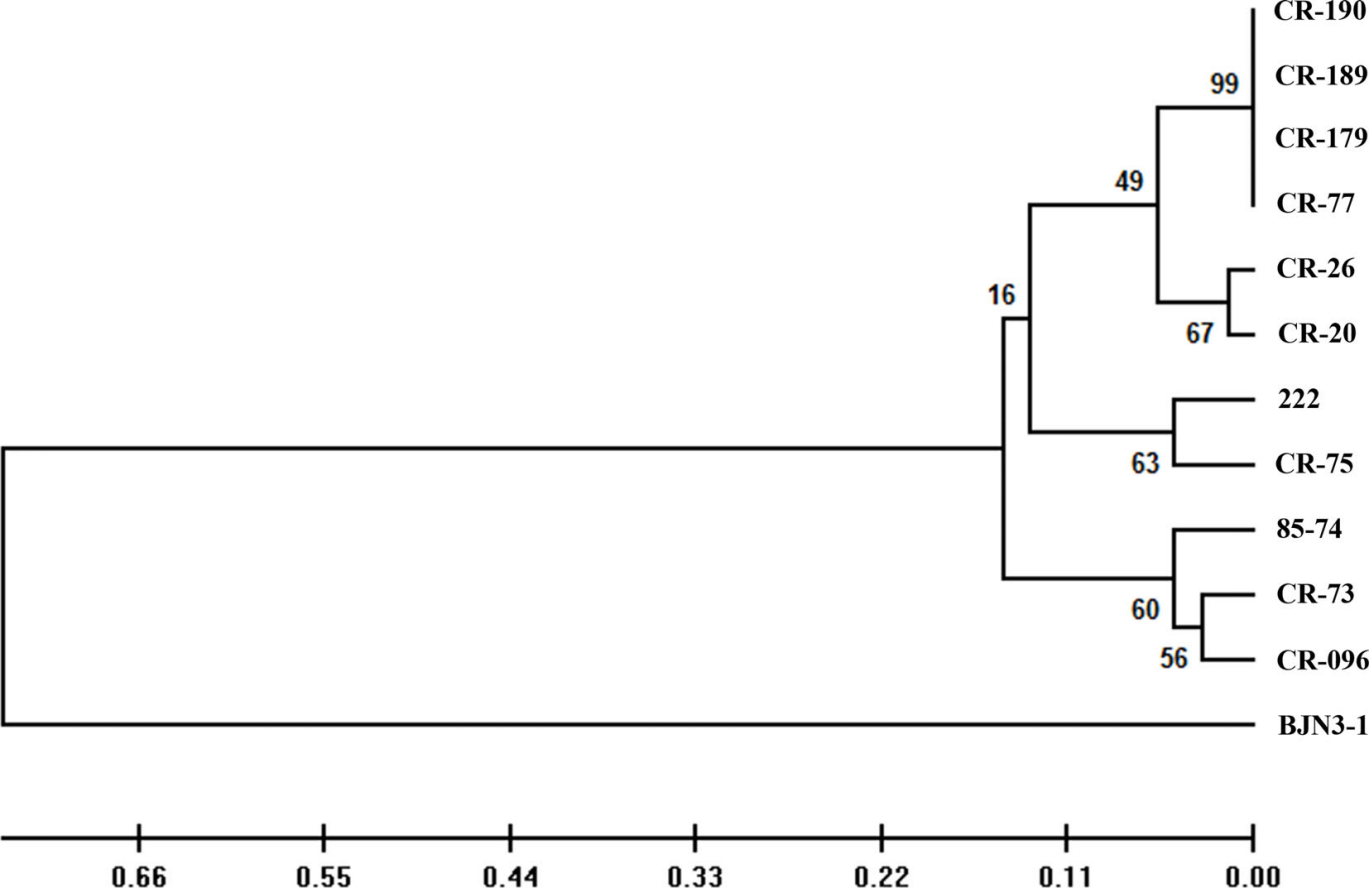
Phylogeny tree showing the clustering pattern of 12 hosts according to the virulence pattern of 37 *Plasmodiophora brassicae* isolates. The scale bar indicates the evolutionary distance.

Eleven CR inbred lines of Chinese cabbage were infected with the above 37 field isolates of *P. brassicae*, and “BJN3-1” was used as the susceptible control (**Table 1**). All individuals from 11 CR lines and “BJN3-1” exhibiting complete resistance and susceptibility, respectively, to 23 isolates, which were classified as 4 different pathotypes (2, 4, 7, and 11) by Williams’ set. Six isolates could infect 1 or more CR lines, and 8 isolates led to clubbed roots in a few plants in several CR lines. In such cases, the resistance tests were repeated more than twice with corresponding isolates. The same results were obtained, indicating that these field isolates are mixture of different pathotypes. This was confirmed by further infection with inoculum prepared from diseased CR plants to those CR lines that exhibited disease symptoms. Such CR lines showing incomplete resistance were considered resistant if the DI value was lower than 25. All the CR lines appeared completely resistant to pathotypes 2 (according to Williams’ set), except “222,” which was susceptible to 1 isolate, SCXC-60. Pathotypes 7 and 11 could infect “BJN3-1” severely, but not any CR lines. The 11 CR lines showed diverse responses to 28 P*. brassicae* isolates of pathotype 4. Out of 28 isolates, 15 showed avirulence to 11 CR lines; however, the other 13 isolates could infect 1–5 CR lines. Among 11 CR lines, 7 showed different responses to isolates belonging to pathotype 4 of Williams’ set, indicating that these CR lines could be used to differentiate the pathotypes of *P. brassicae*. CR lines “CR-77,” “CR-179,” “CR-189,” and “CR-190” showed the same reactions to 37 isolates and assigned into the same group (**Figure 1**).

### Genotypes of 11 CR Hosts at *CR* Loci

Eleven CR hosts and the susceptible control “BJN3-1” were genotyped using public *CR* locus-/gene-linked markers and newly designed markers listed in **Table S2**. The full length of the *CRa* gene was amplified in “222,” “CR-77” “CR-75,” “CR-179,” “CR-189,” and “CR-190,” indicating the presence of the *CRa* gene in these hosts. “85-74,” “CR-77,” and “CR-73” harbored *CRd*, *Crr1*, and *Crr3*, respectively (Pang et al., 2018). The presence of *Crr1a* was also found in “CR-26” and “CR-20.” Crr2 was detected in “CR-75” and Crr4 was detected in “CR-20” and “CR-73.” However, no known *CR* gene was detected in the host of “CR-096,” indicating the presence of unknown gene/genes in this host.

### Development of the SCD System

**Figure 2:**
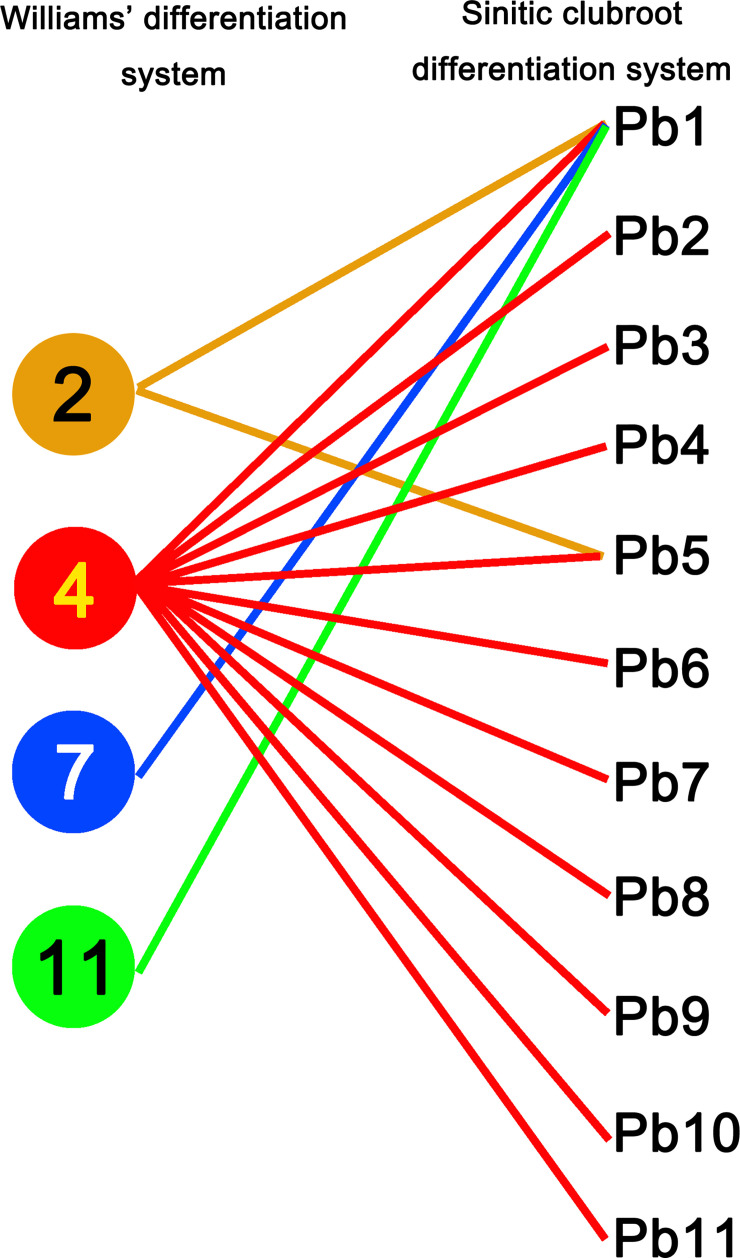
Pathotype comparation of 37 *Plasmodiophora brassicae* isolates identified using Williams’ classification system and the Sinitic clubroot differential set. Numbers 2, 4, 7, 11 on the left indicating the pathotypes classified by Williams’ differentiation system and the Pb1-Pb11 on the right indicating the pathotypes classified by SCD.

The CR lines tested here showed different resistance responses to 37 field isolates of *P. brassicae*, making it possible to select CR hosts for the pathotypic differentiation among them. Since “CR-77,” “CR-179,” “CR-189,” and “CR-190” showed the same response to 37 field isolates and carried the same *CRa* gene, “CR-77” was selected to serve as a representative host for the SCD system development. According to the reaction patterns of eight CR hosts to 37 isolates which were divided into 11 groups, representing the pathotypes from Pb1 to Pb11. Most of the isolates (24 isolates) were classified as Pb1. The pathotypes Pb2, Pb8, and Pb9 have 2 isolates each, and the pathotypes Pb3, Pb4 Pb5, Pb6, Pb7, Pb10, and Pb11 have 1 isolate each. This new clubroot differentiation system was optimally developed with a set of 8 CR hosts (host codes H01–H08) containing known or novel CR gene/genes and 1 clubroot-susceptible host, “BJN3-1” (host code H09), of Chinese cabbage inbred lines (**Table 2**). Twenty-eight field isolates classified as pathotype 4 by Williams’ system were found to have different pathogenicities and were divided into 11 pathotypes by SCD (**Figure 2**, **Table S3**). Those isolates classified as pathotypes 2, 7, and 11 of Williams’ system was reclassified into Pb1 in the SCD system, except for “SCXC-60” was classified as Pb5, a pathotype 2 (**Table S3**). Theoretically, 256 pathotypes can be differentiated by SCD.

### Validation of the Developed SCD System

**Figure 3:**
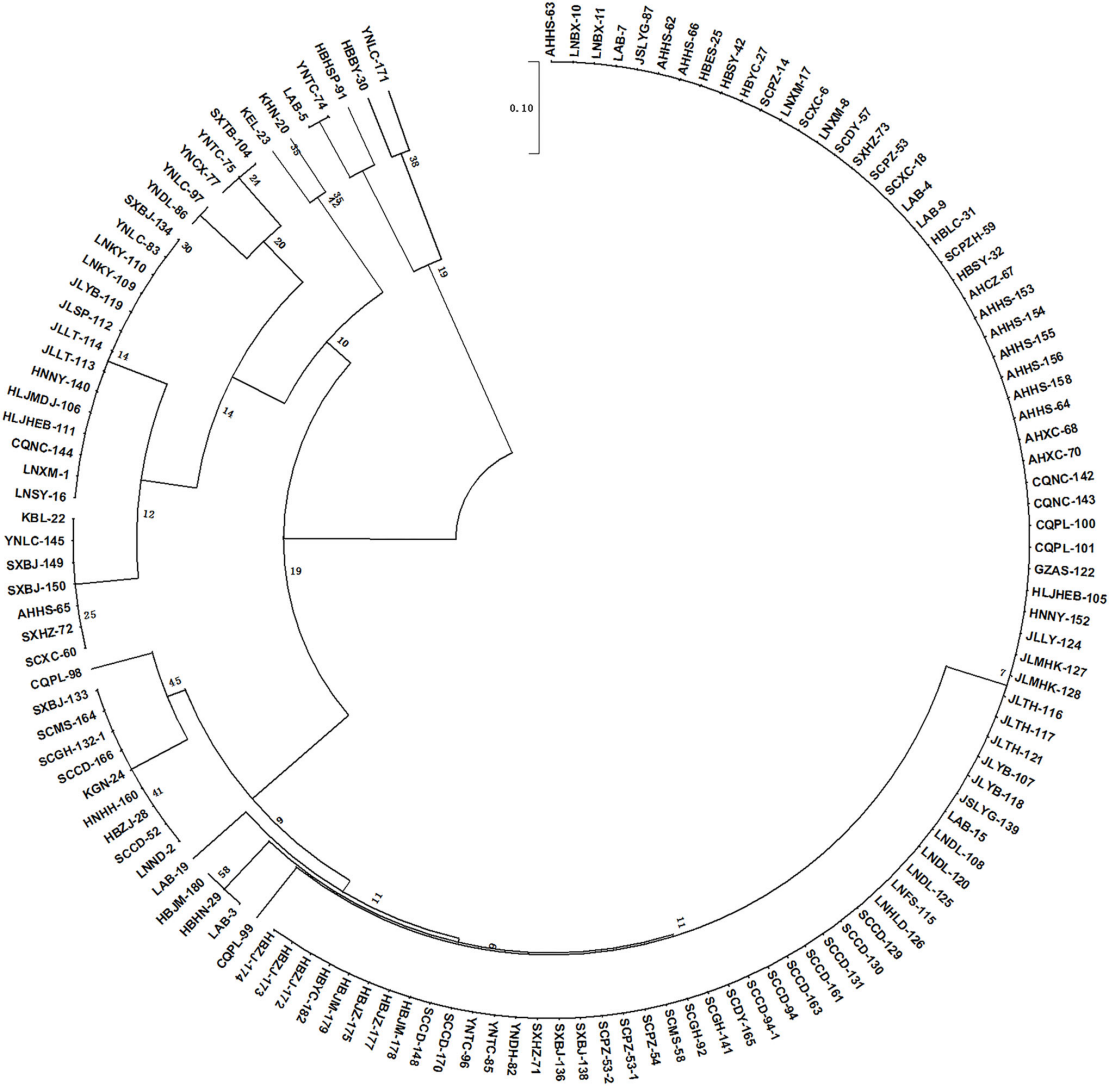
Phylogeny tree showing the clustering pattern of 132 *Plasmodiophora brassicae* isolates according to the developed Sinitic clubroot differential set. The scale bar indicates the evolutionary distance.

To further validate the practicality, stability, and extensibility of the SCD system, a subset of 95 P*. brassicae* field isolates collected from China and Korea were inoculated to the differential hosts of the SCD system. As expected, most of the isolates fell into 11 pathotypes (**Table S3**). Out of 95 isolates, 61 (64%) were found to be Pb1, while 7, 13, 5, 1, 2, and 1 isolates belonged to Pb3, 4, 5, 6, 7, and 8, respectively. Moreover, 3 new pathotypes (Pb12, 13, and 15) were identified from 5 isolates in China, and 2 isolates from Korea extended to the pathotypes 14 and 16 (**Table 2**, **Table S3**). Overall, a phylogenic tree was constructed (**Figure 3**) using 132 isolates and 16 pathotypes were characterized with SCD: 84 (64%) isolates were found to be Pb1, while 9 (7%), 14(11%), and 7(5%) isolates were Pb3, Pb4, and Pb5, respectively. However, only 1 isolate was classified for each pathotype from Pb10 to Pb16. A low pathogenicity of Pb1 prevails in China and appears to be avirulent to all CR hosts harboring single or multiple resistance genes. Pb2 and Pb3 showed virulence to hosts harboring *Crr3* and *CRd*, respectively. Pb11 presented virulence to CR hosts harboring single or multiple resistance genes of *CRa* and *Crr1* but avirulence to hosts harboring *Crr3* or *CRd*. Our results indicated that the SCD system is suitable for the pathotype classification of *P. brassicae* from *Brassica* crops in China and might be applicable to other countries.

### Distribution of *P. brassicae* Pathotypes in China

In total, 125 field isolates collected from known geographical regions and different *Brassica* crops were further analyzed for their distribution properties. It was found that the pathotype distribution followed certain regularity. Pb1 and Pb4 are widely distributed throughout China, while Pb10 and Pb11, Pb12, and Pb13 were only detected in Hubei and Chongqing, respectively. Among 7 pathotypes, Pb6, Pb9, and Pb15 were found exclusively in Yunnan, where rapeseed, cabbage, Chinese cabbage, and radish are widely cultivated, and where clubroot disease was prevalent and occurred earlier in China. According to the hosts’ origin of each collected isolate, Pb9, Pb14, and Pb16 were found most from Chinese cabbage; Pb7, Pb10, Pb11, and Pb15, from oilseed rape; and Pb12 and Pb13, from tuber mustard. One isolate, “YNTC-75,” collected from infected broccoli, was classified as Pb8, which is found in Chinese cabbage also. Pb1 and Pb4 were found in Chinese cabbage, oilseed rape, and tuber mustard. Pb3, Pb5, and Pb6 were found in Chinese cabbage and oilseed rape.

## Discussion

The rapid spread of clubroot disease is threatening *Brassica* crop production worldwide (Dixon, 2009; Chai et al., 2014). Therefore, economical and effective ways to manage clubroot disease are urgently warranted. Clubroot resistance breeding has proved to be a powerful approach, although crop rotation and fungicide application play a role too (Pang et al., 2018). However, the pathotype variation of *P. brassicae* has led to the loss of resistance in CR cultivars of *Brassica* crops (Kuginuki et al., 1999; Strelkov et al., 2016). In this study, a stable and reliable SCD system consisting of 8 CR hosts of Chinese cabbage was developed. A susceptible line was also included as a susceptible control. In theory, this system can distinguish 256 pathotypes of *P. brassicae*, and here, we identified 16 different pathotypes. These differential hosts with relevant CR genes are useful materials to clarify the pathogenesis of *P. brassicae* and to identify the resistance genes. These lines can also provide important materials for breeding CR varieties of *Brassica* crops, as well as for genetic and molecular biological studies of clubroot resistance.

Plant disease-resistant varieties or lines with genetic purity and stable response to plant pathogens are ideal hosts to establish a stable and reliable differential set to define pathotypes or strains of plant pathogens (Inukai et al., 1994). Over the last few decades, several clubroot differential sets have been developed using CR hosts that originated from *B. rapa* ssp. *rapifera*, *B. oleracea*, and *B. napus* or F_1_ hybrids of Chinese cabbage. Among them, two differential sets of Williams and ECD (Williams, 1966; Buczacki et al., 1975) are commonly used by clubroot experts working on clubroot resistance breeding and *P. brassicae*. Among CR hosts, “Badger shipper,” “Jersey Queen,” “Laurentian,” and “Wilhelmsburger” are adopted in Williams’, ECD, and CCD sets (Strelkov et al., 2018). However, we found that intermediate and fluctuating disease scores of most of isolates were often observed in these hosts even when inoculation was performed several times and the susceptible control was fully infected. This leads to difficulties in distinguishing the pathotypes. This phenomenon was also found in other studies (Kuginuki et al., 1999; Kim et al., 2016; Strelkov et al., 2018). However, 8 CR hosts used in the SCD set are inbred lines of Chinese cabbage, and they showed consistent resistance or susceptibility to the field isolates evaluated in this study. A few individual plants showed severe susceptibility to some isolates, although all the “BJN3-1” plants were diseased. This might be attributed to the virulent pathotype present in a small load in the fields. The presence of a virulent isolate was confirmed by the fact that the isolate from diseased plants of CR hosts was compatible with the corresponding CR host. The mixture of more than 2 pathotypes has been reported by Strelkov et al. (2018). In addition, commercial CR F_1_ hybrids of Chinese cabbage have been used for developing clubroot differential systems in Japan and Korea (Kuginuki et al., 1999; Kim et al., 2016). These F_1_ cultivars showed a consistent uniform response to *P. brassicae*. The possibilities of seed contamination with selfed seeds of a susceptible parent may result in an intermediate disease score. There is also a limitation for sustainable use when the commercial F_1_ hybrids are not always available if the demand is shrinking or disappearing within a few years.

Resistant varieties with known resistance genes will provide a great opportunity to develop sets of differential hosts for pathogen differentiation and to breed resistant cultivars. For example, several differential sets with single or multiple characterized resistance genes have been developed for pathotype classification of *M. oryzae* and are widely used for improving rice blast resistance (Atkins et al., 1967; Kiyosawa, 1984; Wu et al., 2004; Shi et al., 2015; Zhang et al., 2017; Jiang et al., 2019). Identification of the *CR* gene or loci in *B. rapa* allowed the evaluation of CR germplasm using *CR* genes or their linked markers. The CR hosts adopted in the SCD system contained either known (such as *CRa*, *Crr1*, *Crr3*, and *CRd*) or novel CR genes. Several CR hosts (H04, H05, and H06) showed different responses to some pathotypes (Pb5, Pb6, Pb9, Pb10, Pb12, Pb13, and Pb15), and even though they shared the same *CRa* gene. Similarly, both H02 (*Crr1* and *Crr4*) and H03 (*Crr1*) harbored *Crr1* but showed different responses to Pb7, Pb14, and Pb16. The different reactions suggest the presence of novel *CR* genes in these hosts. *CR* genes appeared to confer pathotype-specific resistance to *P. brassicae* (Fuchs and Sacristán, 1996; Hasan et al., 2012), leading to the pathogenic variability of *P. brassicae* during the cultivation of uniform CR cultivars, subsequently causing the breakdown of the resistance. To efficiently manage clubroot disease, the selection and cultivation of suitable CR varieties is a pragmatic approach in the region of interest after the pathotype of *P. brassicae* is defined with the SCD system. Differential hosts, besides *B. rapa* in this study, can be used to improve other *Brassica* crops by marker-assisted breeding, such as *B. napus* (Bradshaw et al., 2008; Hirani et al., 2016) and *B. oleracea* (Chiang and Crete, 1983). However, further work is needed to identify new *CR* genes present in these hosts for gene pyramiding.

Since clubroot disease is prevalent in *Brassica* crops worldwide, the developed clubroot differential system should be applicable to differentiate the isolate from different hosts and regions. The isolates tested in this study were collected from different *Brassica* crops and regions of China and Korea. The SCD system could successfully differentiate them into 16 pathotypes. Moreover, 28 isolates classified as pathotype 4 by Williams’ system showed variable reactions when inoculated to SCD hosts and were reclassified into 11 pathotypes by the SCD system. This would be an indication that Williams’ set does not distinguish seemingly different isolates. Additionally, 15 field isolates were classified as pathotypes 2, 4, 7 10 and 11 using Williams differential set in China (Shen et al., 2009, in Chinese). The typical pathotypes of 2(LNBX-10), 4(LNXM-17 and LNXM-8), 7(SCPZ-14), 10(SCXC-6), and 11(LNSY-16) were kindly provided by Prof. Xiangqun Shen and these isolates were reclassified by William differential set (**Table S3**). Pathotypes 7(SCPZ-14), 10(SCXC-6) and 11(LNSY-16) were identified as pathotype 4 in this study (**Table S3**). Therefore, a set of differential hosts for the stable characterization of *P. brassicae* pathotypes is very important for pathological studies and for the improvement of clubroot resistance. It is believed that the coevolution of plants and their pathogens leads to the emergence of new virulence (Fredua-Agyeman et al., 2018). New virulent genotypes of *P. brassicae* might be identified if more isolates are studied and may appear with the erosion of resistance in CR cultivars. The CCD system detected 17 pathotypes with differential hosts from 106 populations of *P. brassicae* in Alberta (Strelkov et al., 2018). Meanwhile, in theory, the SCD system has the potential expansibility to detect as many as 256 pathotypes. Moreover, we successfully tested the applicability of the SCD system using isolates from different *Brassica* crops (Chinese cabbage, oilseed rape, broccoli, and tuber mustard) and different countries (China and Korea).

In this study, considerable diversity in the virulence of *P. brassicae* was observed south of Qinling Mountains–Huaihe River Line, a geographical boundary between the northern and southern regions of China. It belongs to the subtropical zone, with monsoon climate, and diverse *Brassica* crops are grown at different seasons in a year. For example, 7 pathotypes were identified in Yunnan Province, which is the most seriously affected region in China, and 4 or 5 pathotypes were detected in Sichuan, Chongqing, and Hubei Provinces. Interestingly, 5 pathotypes distributed in Shanxi Province belong to the south of China. In these regions, clubroot disease was first observed in 1962 (Wang, 1962), after which an outbreak occurred at the end of the 1990s (reviewed by Chai et al., 2014). Continuous cropping of *Brassica* crops and the rice-rape rotation were widely employed in these regions. However, only 2 pathotypes (Pb1 and Pb4) were observed in the northern regions of China, including Henan, Liaoning, Jilin, and Heilongjiang Provinces, where Chinese cabbage is mainly grown in autumn. Four isolates from Korea were differentiated into 4 pathotypes (Pb3, Pb5, Pb14, and Pb16) but without the predominant pathotype of Pb1, unlike China. Chinese cabbage has been affected by clubroot disease in Korea since 1928 (Cho et al., 2003). The long period of cultivation with CR varieties might be another reason for the emergence of new pathotypes. Studies on the geographic distribution of *P. brassicae* pathotypes indicate that pathotype variation is affected by the cropping system, climate conditions, and history of clubroot disease occurrence (Chai et al., 2014).

In summary, the SCD system developed herein showed great potential for pathotype classification within *P. brassicae* populations in China and other countries. Based on the SCD system, pathotype 4 of Williams’ set was differentiated into 11 pathotypes, and a total of 16 pathotypes were identified from 132 field isolates. Among them, Pb1 was found to be the most predominant pathotype in China. A larger number of virulence groups by a powerful pathotype classification may help us gain a better understanding of the pathogenicity variation of *P. brassicae* in China and further serve as a complementary method to develop other differential systems. This differential set will be helpful for understanding the classification, distribution, variation, and prevalence of *P. brassicae* populations, which in turn should greatly accelerate the breeding of CR Chinese cabbage and other *Brassica* crops.

## Data Availability Statement

All datasets presented in this study are included in the article/**Supplementary Material**.

## Author Contributions

WP performed the experiments, data analysis and drafted the manuscript. YL helped to draft the manuscript. XL and ZZ helped the data analysis and experiments. ZP conceived the study, participated in its coordination, and helped to draft the manuscript. All authors contributed to the article and approved the submitted version.

## Funding

This study was supported by grants from the National Key Research and Development Program of China (2016YFD0100202-19), National Natural Science Foundation of China (CN) (31772326), the earmarked fund for China Agriculture Research System (CARS-12) and the China Postdoctoral Science Foundation (2016M600214).

## Conflict of Interest

The authors declare that the research was conducted in the absence of any commercial or financial relationships that could be construed as a potential conflict of interest.
